# Sleep disturbances in nurse managers during the early and late stages of the COVID-19 pandemic

**DOI:** 10.3389/fpsyg.2022.1070355

**Published:** 2022-12-16

**Authors:** Ángel Boned-Galán, Nieves López-Ibort, Ana Gascón-Catalán

**Affiliations:** ^1^Hospital Universitario Miguel Servet, Zaragoza, Spain; ^2^GIIS092—Liderazgo Relacional en Cuidados de la Salud, Instituto de Investigación Sanitaria de Aragón, Zaragoza, Spain; ^3^Hospital Clínico Universitario Lozano Blesa, Zaragoza, Spain; ^4^Facultad de Ciencias de la Salud, Universidad de Zaragoza, Zaragoza, Spain

**Keywords:** COVID-19, nurse manager, sleep, stress, nursing, hospital, pandemic

## Abstract

**Introduction:**

Working conditions during the COVID-19 pandemic have affected health professionals’ quality of sleep. To date, most of the studies that assess the impact of the COVID-19 pandemic on sleep have been carried out with front-line health personnel, and almost none of them have been carried out with managers.

**Objective:**

To evaluate the quality of sleep and the level of stress in nurse managers during the early and late stages of the COVID-19 pandemic.

**Methods:**

Cross-sectional studies were carried out at two time points: after the lockdown period (July 2020) and a year and a half after the start of the pandemic (October 2021). A total of 102 nurse managers of a tertiary hospital were invited to participate. The Perceived Stress Scale (PSS-14) and Pittsburgh Sleep Quality Index (PSQI) were administered to assess stress levels and sleep quality.

**Results:**

The response rate was 85.2% in 2020 and 81.3% in 2021. Nursing managers showed alterations in sleep quality throughout the pandemic: 70.1% after confinement and 61.4% at the beginning of the second wave. In addition to stress, the fear of contagion by COVID-19 influenced sleep problems. More than a year after the start of the pandemic, the professionals’ fear of contagion decreased. Perceived stress also decreased, but sleep disturbances remained.

**Conclusion:**

High stress and poor sleep quality among nurse managers require special attention, and specific interventions need to be implemented. Hospitals should develop programs that help nurse managers develop skills to mitigate stress levels and thus improve sleep quality and professional quality of life.

## Introduction

COVID-19 appeared in early December 2019 in Wuhan, Hubei Province, China, where the first cases of atypical pneumonia caused by a new coronavirus (SARS-CoV-2) were diagnosed. Neither society nor health systems and organizations considered the possibility of experiencing a pandemic in their planning and health care strategies. For this reason, at the beginning of the pandemic (March 2020), attention to COVID-19 had an unprecedented impact on the Spanish health system. The response of the hospitals to this situation caused them to completely transform. Separate COVID-19 and non-COVID-19 units were created, additional ICU beds were made available, the diagnostic capacity of the laboratories was increased, and nursing units and medical services were relocated to be able to safely care for COVID-19 patients who were isolated from the rest of the patients. At the same time, new work protocols were developed and implemented based on the scientific evidence that was gradually published regarding the pandemic. In Spain, the population was confined for months until hospital pressure had decreased (June 2020). Subsequently, the infection evolved in several waves with lower intensity and less pressure on hospitals. Vaccination of the population, especially vulnerable people and health professionals, marked a turning point, reducing the number of cases and their severity. In October 2021, 79% of the population in Spain was fully vaccinated (Ministerio de Sanidad, 2021).

Nurse managers mainly led the transformation process in hospitals, both in the organization as whole and nursing units in particular, probably facing the most complicated management tasks of their managerial careers.

The role of nurse managers is essential in the health environment to guarantee a healthy work environment, influencing organizational performance, nurses’ satisfaction and empowerment, staff turnover, and patient outcomes (Udod, 2014; Al Maqbali, 2015). However, the working conditions generated during the COVID-19 pandemic meant that supervisors faced an unknown and hostile hospital work environment, with a shortage of medical materials and personal protective equipment, excessive work overload, time pressure, a lack of nurses (in number and training), long working hours, a risk of exposure to infections, a lack of information, separation from their families (due to schedules and fear of contagion), and sometimes a lack of social support (Tu et al., 2020; Zhao et al., 2020). In addition, nurse managers experienced a large number of hospital admissions, the rapid deterioration of patients’ physical conditions, patients dying without family at their bedside, the fear of not being prepared to care for COVID-19 patients, and a new feeling of helplessness as they learned to cope with this unknown disease (American Nurses Association, 2020).

In these circumstances, health professionals may have been one of the main groups that suffered from sleep disorders. Sleep quality is a complex phenomenon that conceptually includes quantitative aspects such as sleep duration, sleep latency or the number of awakenings, and qualitative aspects such as ease of falling asleep, sleep maintenance, anxiety when trying to sleep, early awakenings, movements during sleep, tension, agitation during the night, and the perception of the depth of sleep (Demir Zencirci and Arslan, 2011).

Alterations in sleep quality are frequent in the adult population. One study showed that one-third of adults in Western countries experienced difficulty falling asleep or staying asleep at least once a week (Leblanc et al., 2009). The American Academy of Sleep Medicine has stated that 30–35% of the adult population in the United States has symptoms of insomnia (Deng et al., 2020). The incidence of sleep disorders among the Asian population in general ranges from 26.4–39.4% (Wong and Fielding, 2011).

In nurses, the figures related to sleep problems are the same or higher than those in the general population. The incidence of insomnia among nurses in a region of Poland was 47.8% (Zdanowicz et al., 2020). An investigation showed that 35.5% of Chinese nurses suffered from sleep disorders (Liu, 2015). Another study in four tertiary hospitals in Shanghai reported an average incidence of insomnia of 57.4% among nurses (Yang et al., 2011). Several authors maintain that these sleep disturbances are likely due to the peculiarity of their work, which includes great responsibility, a heavy workload, great pressure, the need to work in shifts (Huang et al., 2018), a high level of job stress (Thichumpa et al., 2018), high demands, and complex work environments (Liu et al., 2019).

Poor sleep quality among hospital nurses is a critical problem for the health system because it could have serious consequences both for nurses and for their patients. In nurses, poor sleep quality can lead to hypertension, exhaustion, and depression (Liu et al., 2016; Giorgi et al., 2018) and even an increased tendency to commit suicide (Mieda and Sakurai, 2013). Regarding patient care, safety can be compromised, and the quality of care can be reduced due to the greater probability that nurses will have poor work performance (Zhou et al., 2020). At the same time, impaired sleep quality is related to a decrease in the level of concentration and greater distraction, which leads to increased occupational risks, absenteeism (Kucharczyk et al., 2012; Uehli et al., 2014), and nurse turnover intentions (Søbstad et al., 2021).

Previous studies suggest that job stress may be a factor that directly correlates with poorer sleep quality in nurses (Han et al., 2016). High demands at work and a heavy workload can cause sleep disorders (Yazdi et al., 2014), but stressors in the work environment are the most common causes of sleep problems (Udod et al., 2017).

Stress is a complex multidimensional phenomenon that is determined by a person’s subjective perceptions and interpretations and may be evaluated as damage, loss, threats, or challenges. Stress manifests as a biopsychosocial response when an individual’s perceptions of the work environment are considered demanding or to exceed their resources, endangering their well-being (Udod et al., 2017).

The pandemic has increased and exacerbated all the factors that have been identified in previous studies, under normal conditions, as the main causes of stress among nursing managers (Labrague et al., 2018). Warshawsky and Havens (2014) recognized the stressful role of the nurse manager position due to physical labor, long work hours, staffing, and interpersonal relationships (Warshawsky and Havens, 2014).

Recent studies show that stress is a significant problem, especially among nursing unit managers (Labrague et al., 2018). McCallin and Frankson concluded in their study that the nursing manager position is complex, multifaceted, and challenging (McCallin and Frankson, 2010). The 2003 Canadian Community Health Survey showed that two-thirds (67%) of head nurses and nurse managers reported high work stress, among the highest of the health care occupations (Statistics Canada, 2007).

Persistent exposure to stress negatively affects not only the health of nurse managers but also their decision-making processes, which can potentially affect staff, patient and organizational outcomes (Shirey et al., 2013).

Therefore, all these drivers of stress (increased and exacerbated stress, as discussed above) have been challenging for nursing managers as they have tried to manage the pandemic in their units. They have the burden of managing the crisis not only operationally but also mentally, emotionally, and even ethically (Dimino et al., 2021). Consequently, it would be expected that this work role, altered by the COVID-19 pandemic, would have an impact on the occupational well-being of nursing managers, specifically on the quality of sleep and the level of work stress.

Therefore, understanding this situation is important due to the adverse effects of poor sleep quality and stress on nurse manager health, staff satisfaction, and patient outcomes (Shirey et al., 2010). However, to date, little is known about the impact of the current crisis on psychological well-being over time, specifically on sleep quality and stress, among nurse managers, who have had to manage this unprecedented crisis in Spanish hospitals. Therefore, our objective was to evaluate the quality of sleep and the level of stress among nurse managers during the early and late stages of the COVID-19 pandemic.

## Methodology

### Design

Cross-sectional studies were carried out at two time points: after the lockdown period (July 2020) and a year and a half after the start of the pandemic (October 2021).

### Population/sample

The participants were nurse managers of a tertiary hospital. This hospital employed 102 nurse managers, 1,806 registered nurses, and 1,324 assistant nurses. The study population was the same at both time points except for the nurse managers who changed workplaces or retired.

All nurse managers who were working at the time the study was conducted were invited to participate. All nurse managers who answered the questionnaire and accepted the conditions of informed consent were included. There were no exclusion criteria. Of the 102 nurse managers who received the questionnaire, 87 completed it in 2020, and 83 completed it in 2021. There were no questionnaires with missing data, so all answered questionnaires were valid for inclusion in the study.

### Procedure

The invitation to participate in the study was sent by email from nursing management and included the link to the online form. A period of 30 days was established for completion of the form. Another email was sent after 15 days reminding the participants of the deadline. The online questionnaire was designed using the Google platform. It included a paragraph informing the participants of the objective of the research, as well as that by sending the questionnaire, informed consent was provided. Anonymity was guaranteed since no personal data were collected that would allow the unequivocal identification of those who decided to participate.

### Variables and measurement instruments

The following sociodemographic and labor variables were collected: age, sex, marital status, years of experience as a nurse manager, and type of shift (morning or rotating).

The level of fear of COVID-19 was assessed using an analog scale scored from 0 to 10, where higher values indicated a higher level of fear.

The Perceived Stress Scale (PSS-14) is used to assess the level of stress perceived during the last month (Cohen et al., 1983). It is a self-administered questionnaire with 14 items. Each item is scored from 0 to 4 (0 = never; 1 = almost never; 2 = sometimes; 3 = fairly often; 4 = very often). The total score is obtained by reverse scoring Items 4, 5, 6, 7, 9, 10, and 13 and then summing the scores across all 14 items. Total scores range from 0 to 56. The higher the score, the higher the level of perceived stress. As it is not a diagnostic element, there are no cutoff values, and this was agreed upon by the researchers. Cronbach’s alpha in this study was 0.90 in 2020 and 0.89 in 2021.

To objectively measure sleep quality, polysomnography is usually used as the gold standard. The drawbacks that this technique presents have led to the development of other subjective instruments for sleep quality measurement, such as the Pittsburgh Sleep Quality Index (PSQI; See et al., 2007). The PSQI questionnaire is a widely used, multidimensional self-report measure of sleep quality and disturbances over a 1-month time interval. Therefore, this questionnaire was used to assess sleep quality in this research. The 19 items of the questionnaire generate scores for seven components: subjective sleep quality, sleep latency, sleep duration, habitual sleep efficiency, sleep disturbance, the use of sleeping medication, and daytime dysfunction. The component scores range from 0 (indicating no difficulty) to 3 (indicating severe difficulty). The sleep component scores, referred to as the global score, are summed to yield a total score ranging from 0 to 21, with a higher total score indicating worse sleep quality. A cutoff point of 5 is used; if a person exceeds this score, their sleep quality is considered to be poor (Buysse et al., 1989). As this value increases, a subject’s sleep quality decreases (Satizábal and Marín, 2018). In this study, Cronbach’s alpha was 0.80 in 2020 and 0.84 in 2021.

### Data analysis

A descriptive analysis of the data, percentages, and frequencies for the qualitative variables and the mean and standard deviation for the quantitative variables was carried out. Chi-square tests were used to compare sociodemographic and work data at two time points: the early and late stages of the COVID-19 pandemic. The Kolmogorov–Smirnov test was used to evaluate whether the variables followed a normal distribution. To study whether there were differences between groups, the nonparametric Mann–Whitney U test (two groups), or the Kruskal–Wallis test (more than two groups) was used. Spearman’s correlation coefficient was used to analyze the linear relationship between the Pittsburgh global sleep quality index, the level of perceived stress, and the fear of contagion by COVID-19. Differences were considered statistically significant when *p* < 0.05.

### Ethics statement

The design of the questionnaire for the collection of sociodemographic data guaranteed the complete anonymity of all the participants. All participants gave their informed consent when completing the survey. This study was approved by the Aragón Ethics Committee, CEICA (n° 16/2020 C.P. - C.I. PI20/371).

## Results

In 2020, 87 of the 102 nurse managers responded to the questionnaire (85.2%), and in 2021, 83 of the 102 responded (81.3%).

The sample mainly consisted of married women aged between 36 and 55 years who worked morning shifts. Approximately half of the sample had been working as a nurse manager for less than 5 years. There were no significant differences in terms of sociodemographic and labor variables between 2020 and 2021 (Table 1).

**Table 1:** Sample characteristics through the study years of 2020 and 2021.

Variables	Category	2020 *N* (%)	2021 *N* (%)	*p*
Age	≤35	6 (6.9)	3 (3.6)	0.697
	36–45	33 (37.9)	34 (41)	
	46–55	28 (32.2)	30 (36.1)	
	≥55	20 (23)	16 (19,3)	
Sex	Female	75 (86.2)	69 (83.1)	0.671
	Male	12 (13.8)	14 (16.9)	
Marital status	Single	13 (14.9)	12 (14.5)	0.989
	Married	63 (72.4)	61 (73.5)	
	Has a partner	6 (6.9)	6 (7.2)	
	Divorced/Separated	3 (3.4)	3 (3.6)	
	Widowed	2 (2.3)	1 (1.2)	
Shift	Morning	74 (85.1)	70 (84.3)	1.000
	Rotating	13 (14.9)	13 (15.7)	
Years as nurse manager	< 5	46 (52.9)	45 (54.2)	0.624
	5–15	29 (33.3)	23 (27.7)	
	>15	12 (13.8)	15 (18.1)	

The sleep quality of nurse managers during the COVID-19 pandemic, based on the PSQI global score, was similar at the two time points of the study. The mean (SD) values obtained were 8.45 (4.29) in 2020 vs. 8.18 (5.04) in 2021. A small decrease in the mean was observed, but this difference was not statistically significant (*p* = 0.71). A total of 70.1% of the study participants in 2020 and 61.4% in 2021 were classified as poor sleepers (PSQI global scores >5).

No significant differences were observed in the quality of sleep with respect to the sociodemographic characteristics for age, sex, or marital status at the two time points of the study (early or late stage of the pandemic; Table 2).

**Table 2:** Associations between sleep quality (Pittsburgh Sleep Quality Index global score) and sociodemographic and labor variables.

		2020 Mean (SD)	*p*	2021 Mean (SD)	*p*
Age	≤35	11.83 (2.92)	0.124	14.00 (5.29)	0.106
	36–45	8.52 (3.91)		7.56 (4.91)	
	46–55	7.36 (4.38)		8.93 (5.38)	
	≥55	8.85 (4.72)		7.00 (3.93)	
Sex	Female	8.48 (4.36)	0.864	8.17 (4.88)	0.978
	Male	8.25 (3.98)		8.21 (5.92)	
Marital status	Single	7.08 (4.49)	0.292	9.17 (5.09)	0.625
	Married	9.06 (4.25)		8.18 (5.14)	
	Has a partner	7.00 (3.03)		6.00 (4.73)	
	Divorced/Separated	5.67 (4.50)		10.00 (3.60)	
	Widow	6.50 (6.36)		4.00	
Shift	Morning	8.77 (4.21)	0.095	8.83 (4.99)	0.006
	Rotating	6.62 (4.38)		4.69 (3.75)	
Years as nurse manager	< 5	8.89 (4.17)	0.561	9.04 (5.08)	0.049
	5–15	7.79 (4.45)		6 (4.23)	
	>15	8.33 (4.49)		8.93 (5.33)	

The mean score for sleep quality was very similar when comparing the data of the nurse managers who worked morning shifts at the two study time points. In the case of nurse managers who worked rotating shifts, the mean value was lower after more than a year of the pandemic (late stage), indicating a better quality of sleep in these nurse managers.

Nurse managers who worked morning shifts had poorer sleep quality than those who worked rotating shifts. This difference was only significant in 2021 (*p* = 0.006).

Table 3 describes the sleep characteristics of supervisors during the pandemic. The average number of hours they slept during the pandemic was below 6 h per day: 5.47 h in 2020 and 5.80 h in 2021. There was a slight increase in sleep time in 2021, although it was not significant. Only 16.1% of supervisors (*n* = 14) in 2020 and 21.7% of supervisors (*n* = 18) in 2021 slept at least 7 h per day (*p* = 0.231).

**Table 3:** Sleep quality, stress and fear of contagion by COVID-19 throughout the COVID-19 pandemic (2020 and 2021).

	2020 Mean (SD)	2021 Mean (SD)	*p*
Hours of sleep per day	5.47 (1.10)	5,80 (1,02)	0.074
Sleep quality	1.55 (0.79)	1.24 (1.30)	0.060
Sleep latency	1.43 (1.09)	1.05 (1.03)	0.022
Sleep duration	1.49 (0.95)	1.42 (0.75)	0.583
Habitual sleep efficiency	1.01 (1.12)	0.84 (0.97)	0.297
Sleep disturbances	1.48 (0.64)	1.34 (0.57)	0.121
Use of sleeping medication	0.41 (0.86)	0.45 (0.93)	0.815
Daytime disfunction	1.07 (0.79)	1.29 (1.52)	0.234
Global score (PSQI)	8.45 (4.29)	8.18 (5.04)	0.709
Fear of contagion by COVID-19	6.27 (1.93)	5.64 (2.34)	0.054
Stress (PSS-14)	27.07 (8.01)	24.33 (8.16)	0.028

The scores for global sleep quality and for the different components of the scale were similar at the two time points of the pandemic, with no statistically significant differences. The only component in which a significant difference was observed (*p* = 0.02) was sleep latency. The values indicated an improvement, with lower sleep latency 1 year after the start of the pandemic [1.43 (1.09) vs. 1.05 (1.03)].

The fear of contagion by COVID-19 and stress levels was also studied due to their possible effect on sleep quality. The fear of contagion by COVID-19 decreased as time passed since the start of the pandemic, and this difference was almost significant (*p* = 0.054) when comparing the data between 2020 and 2021. The level of stress was higher in the initial months of the pandemic. There was a significant difference in the stress level perceived by the nurse managers when comparing the years 2020 and 2021 (*p* = 0.028).

During the COVID-19 pandemic, a positive correlation was observed between nurse managers’ sleep disorders and stress levels. In other words, sleep problems increased as perceived stress increased, and this was observed both in the initial months and more than a year after the start of the pandemic. However, there was only a positive correlation between sleep problems and the fear of contagion at the start of the pandemic. In 2021, the correlation between these two variables was no longer observed (Table 4).

**Table 4:** Correlations between sleep quality and stress and the fear of contagion by COVID-19 during the pandemic (2020 and 2021).

	Years	Stress	Fear of contagion by COVID-19
		*r* (*p*)	*r* (*p*)
Sleep Quality	2020	0.547 (< 0.001)	0.247 (0.021)
	2021	0.489 (<0.010)	0.128 (0.248)

## Discussion

To our knowledge, this is one of the first studies focused on assessing the perception of sleep quality and stress throughout the COVID-19 pandemic, with only nurse managers as the study population. Most of the research carried out to date has focused on front-line professionals who care for patients.

Our results on the quality of sleep and the stress level of nurse managers in the pandemic are similar to those observed in nurses and other front-line professionals (Ortega-Galán et al., 2020; Tu et al., 2020). This does not agree with the results observed by Simonetti in a study carried out in Italy in the initial phases of the pandemic, where it was concluded that management work was a protective factor against anxiety and sleep disturbances, as managers did not work directly with COVID-19 patients (Simonetti et al., 2021). Although it is true that, as a general rule, nurse managers do not have direct contact with patients, their responsibilities and the leadership of their teams in a situation as uncertain and unknown as the current pandemic may have contributed to increased stress and sleep disturbances. At the beginning of the pandemic, the main stressors were ignorance of the disease, the fear of contagion, the lack of individual protection equipment, the turnover and lack of training of many professionals, work overload, and constant changes in the organization of hospitals to adapt to hospital admissions (Tan et al., 2020; Murat et al., 2021; Zheng et al., 2021). Currently, in Spain, work overload is still maintained due to the shortage of nurses being hired. All these factors have affected both frontline staff and other professionals, regardless of the unit in which they work (Zarzour et al., 2021).

This work overload may have been a factor that has contributed to the poor sleep quality of nurse managers during the pandemic. Our data show that nurse managers do not have good sleep quality and that sleep quality has not changed substantially as the pandemic has progressed. The results observed in 2020 (8.45 ± 4.29) and in 2021 (8.18 ± 5.04) showed minimal improvement in the sleep quality of the nurse managers, a difference that was not statistically significant. When our data were compared with a study carried out in nurses working in hospitals in our country, global PSQI values showed better sleep quality before the pandemic (Gómez-García et al., 2016).

Poor sleep quality was observed in the entire sample, with no differences regarding age, sex, or marital status. Some studies have reported poorer sleep quality in women (Lai et al., 2020; Luo et al., 2020; Simonetti et al., 2021) or in single people due to less social support (Al Maqbali, 2021).

However, differences were observed with respect to the shift performed; nurse managers who worked rotating shifts had better quality of sleep. This does not agree with the results of other studies (Kim-Godwin et al., 2021; Zheng et al., 2021) which could be explained by the fact that our data are for nurse managers and not for care nurses.

Nurse managers who work rotating shifts perform general supervision functions, resolving incidents that occur in the hospital during their work shift and passing the baton to nurse managers who work morning shifts. The latter group has one or more units assigned to them, being responsible, among other aspects, for their human and material resources. In our country, the problem of the availability of personal protective equipment, which existed at the beginning of the pandemic in 2020, has been solved, but the same has not happened in hiring nurses to cover absenteeism. This means that morning shift nurse managers must dedicate a greater amount of time and effort to keep their units running, even extending their work hours beyond those established. This can increase their worries and decrease their ability to disconnect when they leave the hospital, which can influence the quality of their sleep. Likewise, participants who had been working as nurse managers for a shorter time presented a worse quality of sleep. The most plausible explanation is that the experience acquired during the years of working as a nurse manager acted as a protective factor by reducing anxiety and thus favoring a better quality of sleep (Huang and Zhao, 2020; Lai et al., 2020). This result was expected because to manage crisis situations, it is equally important to have adequate training and work experience in the position, which in turn provides greater self-confidence (Warshawsky and Cramer, 2019).

Another noteworthy aspect was that a decrease was found in the evolution of the fear of contagion to COVID-19 between the two time points of the study; however, this difference was not statistically significant. In addition, it was observed that the fear of contagion was correlated with a worse quality of sleep only in 2020, showing a trend toward the normalization of the pandemic.

When analyzing the different components of the PSQI scale, we wanted to highlight the number of hours of sleep per day and sleep latency. Regarding the number of hours of sleep per day, although the number increased slightly compared to that at the start of the pandemic, it is far from the consensus on the hours of sleep that an adult should get to achieve healthy sleep, which is between 7 and 9 h (Merino Andréu et al., 2016). The following consequences of short sleep duration have been described in the literature: daytime fatigue, psychomotor impairment, accidents, the deterioration of physical and psychological health, and poor academic or work performance. Sleep latency and its decrease when compared to that in the initial stage of the COVID-19 pandemic could be related to the observed decrease in stress levels throughout the pandemic, as shown by the data from our research and other studies (Kim-Godwin et al., 2021).

Previous research has pointed to stress as a predictor of sleep quality (Deng et al., 2020; Salari et al., 2020; Al Maqbali, 2021; Kim-Godwin et al., 2021), even more so when it occurs in the context of a pandemic and the population studied includes health professionals (Maunder et al., 2006). The level of perceived stress at the beginning of the pandemic was higher than that observed before the pandemic in 2019, when our data were compared with a study carried out in the same population of nurse managers and in the same hospital as the present study (Boned Galán, 2019).

During the pandemic period, a statistically significant decrease was observed, with values of 27.07 ± 8.01 in 2020 (early COVID-19) and 24.33 ± 8.16 in 2021 (late COVID-19), which indicates that nurse managers’ stress is reducing as the pandemic progresses.

A year and a half after the start of the pandemic (2021), the values of perceived stress were quite close to those observed before the pandemic. This fact has already been observed in previous pandemics and can be attributed to the progressive adaptation to stressors, as the situation is perceived in a more favorable way (Su et al., 2007). Other aspects that should be taken into account include greater knowledge about the disease, the current availability of personal protective equipment, the effectiveness of a vaccine in reducing the severity of cases, reduced fear of contagion, and a decrease in health care pressure in hospitals related to the pandemic.

The WHO has highlighted the need to keep all staff protected from chronic stress and poor mental health from the start of the pandemic. It is evident that the quality of sleep is poor not only during the pandemic, but it is also a chronic problem that should be addressed in nursing. Additionally, nurse managers may experience additional pressure related to the functions derived from their role. Hospitals should develop strategies that support nurse managers, providing training in soft skills and management. Personalized care programs for these health professionals should include psychological support, team building activities, mentoring or coaching programs adapted to the needs and maturity of the work teams and the individuals that make them up. Likewise, the underlying organizational factors that influence their role must be addressed. Span of control of the nurse manager should be adapted, considering aspects related to the characteristics of the unit, the professionals working in it, and the organization itself. In addition to developing human resources policies aimed at maintaining an appropriate nurse–patient ratio in care units considering the levels of dependency and complexity of patients. These managers should act as role models regarding care to mitigate stress (World Health Organization, 2020).

### Limitations

A limitation is that only one hospital was included in the current study. Thus, the results may not be generalizable to the experiences of nurse managers in other hospitals.

Because participation in the study was voluntary, it is possible that the respondents differed from the nonrespondents in some meaningful way (Enders, 2006). Nevertheless, the response rate was high (85.2 and 81.3%), which minimizes concerns about the representativeness of the respondents.

The use of self-reports may also have been an important limitation if we compare it with the use of other means, such as clinical interviews or functional neuroimaging, due to social desirability bias.

Unfortunately, a causal relationship between the pandemic and the observed sleep disturbances could not be established, due to the absence of data on sleep quality in this sample of nurse managers before the COVID-19 pandemic.

## Data availability statement

The raw data supporting the conclusions of this article will be made available by the authors, without undue reservation.

## Ethics statement

The studies involving human participants were reviewed and approved by the Aragón Ethics Committee, CEICA (n° 16/2020 C.P.-C.I. PI20/371). The patients/participants provided their written informed consent to participate in this study.

## Author contributions

ÁB-G, NL-I, and AG-C: conceptualization, methodology, data analysis, writing—original draft preparation, and writing—review and editing. ÁB-G and NL-I: resources. NL-I: funding acquisition. All authors contributed to the article and approved the submitted version.

## Funding

This research has funded by the Instituto de Investigación Sanitaria de Aragón (IIS Aragón), Zaragoza, Spain.

## Conflict of interest

The authors declare that the research was conducted in the absence of any commercial or financial relationships that could be construed as a potential conflict of interest.

## Publisher’s note

All claims expressed in this article are solely those of the authors and do not necessarily represent those of their affiliated organizations, or those of the publisher, the editors and the reviewers. Any product that may be evaluated in this article, or claim that may be made by its manufacturer, is not guaranteed or endorsed by the publisher.
